# Suppression of high lipid diet induced by atherosclerosis sarpogrelate

**DOI:** 10.1111/j.1582-4934.2012.01554.x

**Published:** 2012-09-26

**Authors:** Yan-Jun Xu, Ming Zhang, Lei Ji, Vijayan Elimban, Li Chen, Naranjan S Dhalla

**Affiliations:** a Institute of Cardiovascular Sciences St. Boniface Hospital Research Department of Physiology Faculty of Medicine, University of ManitobaWinnipeg, MB, Canada; b Department of Pharmacology, Jilin UniversityJilin, China; c Department of Cardiology, Jilin UniversityJilin, China

**Keywords:** sarpogrelate, atherosclerosis, blood viscosity, oxidative stress, high cholesterol diet

## Abstract

Sarpogrelate (SP), a serotonin (5-HT2A) receptor antagonist, is used as an anti-platelet agent for the treatment of some vascular diseases. SP has been reported to inhibit 5-HT induced coronary artery spasm, increase in intracellular calcium and smooth muscle cells proliferation. This study was undertaken to test that SP suppresses the development of atherosclerosis due to high cholesterol diet (HCD) by decreasing blood viscosity and oxidative stress. For this purpose, 29 rabbits were divided into four groups: control group (normal diet); normal diet group with SP at the dose of 5 mg/kg/day; HCD group fed 1% cholesterol; and HCD group with SP at the dose of 5 mg/kg/day. After 90 days of the experiment, blood samples were collected and the animals were killed; the thoracic aorta was stained by the Oil Red O staining method. The results indicate that plasma levels of cholesterol, triglycerides and malondialdehyde were increased in rabbits fed HCD. Plasma viscosity and whole blood viscosity were also higher in the HCD group than that in normal diet group. Treatment with SP prevented these alterations induced by HCD whereas this agent had no significant effect in rabbits fed normal diet. Morphological examination of the aorta revealed that SP treatment prevented the formation of foam cells and atherosclerotic plaque. It is suggested that the beneficial effects of SP in atherosclerosis may be due to actions on blood viscosity, lipid levels and oxidative stress.

## Introduction

5-hydroxytryptamine (5-HT) is important in the regulation of cardiovascular function as its blood concentration was found to be increased in several disease states like stress, hypertension, myocardial infarction and stroke [1]. 5-HT also has been shown to increase heart rate, vascular smooth muscle (VSMC) contraction and cell proliferation [2, 3]. Thus the blockade of 5-HT receptors is considered to be a useful approach for the prevention and treatment of different cardiovascular diseases [4]. In fact, a specific 5-HT2A receptor antagonist, sarpogrelate (SP, Anplag, (±)-1-[2-[2-(3-methoxyphenyl)ethyl]phenoxy]-3-(dimethylamino)-2-propyl hydrogen succinate hydrochloride) has been used clinically as an anti-platelet agent for the treatment of peripheral artery disease and thrombosis [5]. Some experimental studies in rats have revealed that SP has cardioprotective effects in ischaemia-reperfusion induced injury, diabetic cardiomyopathy and myocardial infarction [6–8]. Previous reports from our laboratory have indicated that SP blocked the increase in intracellular concentration of free calcium ([Ca^2+^]_i_) induced by 5-HT and inhibited the proliferation of VSMC [3, 9]. Although Hayashi *et al*. [10] have reported that SP may slow down the progression of atherosclerosis through eNOS pathway, the status of oxidative stress has not been examined in this condition. Furthermore, blood viscosity and plasma viscosity, which are high in hyperlipidaemia, may play a role in the development of atherosclerosis. It may be noted that high blood viscosity has been shown to increase resistance to blood flow, cause damage to vascular endothelial cells, enhance the deposition of lipids in the blood vessel wall and induce the formation of atherosclerotic plaque [11–13]. The present study was therefore undertaken to investigate the effects of SP on blood viscosity, oxidative stress and blood lipids in a rabbit model of atherosclerosis induced by high cholesterol diet (HCD).

## Material and methods

### Materials

The experimental procedures were approved by the Institutional Animal Ethical Committee and approved by the Animal Care Committee of Jilin University, China (Certificate Number: scxk2007-0003). Twenty-nine New Zealand white male rabbits (2.2 ± 0.5 kg) were purchased from the Experimental Animal Holding of Norman Bethune Medical College, Jilin University. Cholesterol was purchased from Amresco Inc., USA. Colorimetric diagnostic kits for fasting total cholesterol (CHOD-POD, cholesterol oxidase peroxidase), and triglycerides (GPO-POD, glycerol-phosphoric acid oxidase peroxidase) were obtained from Beijing BHKT Clinical Reagent Co. Ltd, Beijing, China. Malondialdehyde (MDA) and superoxide dismutase (SOD) test kits were obtained from Nanjing Jiancheng Bioengineering Institute, Nan Jing, China. Sarpogrelate was a gift from Mitsubishi Tanabe Pharma Corporation, Osaka, Japan.

### Methods

Rabbits fed HCD were chosen as an atherosclerotic animal model in the present study. Twenty-nine adult male rabbits (weighing 2.2 ± 0.5 kg) were divided into four groups that were fed a regular chow as control group (*n* = 8), a normal diet plus SP treatment (*n* = 4), a HCD as high lipid diet group (*n* = 12) and a HCD plus SP treatment group (*n* = 5) respectively. The hypercholesterolaemia animals were fed a HCD containing 1% cholesterol for 90 days [14], the drug treatment group fed regular chow containing SP (5 mg/kg/day) for 3 days and then fed a HCD supplemented with SP (5 mg/kg/day) for an additional 90 days. The animals in normal diet plus SP group were given regular chow containing 5 mg/kg/day for 90 days. All animals with or without treatment were used at 90 days for biochemical and morphological analysis.

#### Measurement of blood viscosity and plasma viscosity

The method for blood viscosity measurement was the same as described previously [15]. Briefly, the blood samples were obtained from rabbit ear by vein puncture without anaesthesia under calm state. Anticoagulant blood sample (1 ml containing 900 μl blood and 100 μl 3.8% sodium citrate) was used for whole blood viscosity measurement at 3, 30, 90, 100, 180/S shear rate; 1 ml plasma was also used for plasma viscosity measurement at 100/S shear rate by blood viscometer (BV-100; Beijing Tai Nuode Institute for New Technologies, Beijing, China). The blood viscosity was expressed as centipoise.

#### Measurement of total cholesterol and triglycerides

Rabbits were fasted for 12–16 hrs. Blood was collected from ear vein and plasma was separated by centrifuge at 3500 × *g* for 10 min. Total cholesterol (TC), triglycerides (TG) were measured by using commercially available colorimetric diagnostic kits according to the manufacturer's instructions. Absorbance of standards and samples were determined spectrophotometrically (Sunrise™ Absorbance Reader; Tecan Group Ltd., Mannedorf, Switzerland) at 540 nm. Results were calculated from the standard and were reported as mmol/L.

#### Measurement of MDA and SOD

Plasma SOD (Hydroxylamine method) and MDA (Thiobarbituric acid method) were measured by using commercially available colorimetric diagnostic kits according to the manufacturer's instructions. The blood samples were taken as described above. Absorbance of standards and samples were determined spectrophotometrically (Tecan Sunrise™ Absorbance Reader) at 540 nm. Results were calculated from the standard and were reported as mmol/l.

#### Morphologic examination of atherosclerotic lesions

At the end of the study period, the animals were killed and aortic arch as well as thoracic aorta were removed; photographs were taken after staining with Oil Red O to observe the extent of atherosclerotic plaque in the aortic arch and thoracic aorta. The portion of aortic arch and thoracic aorta were fixed with 10% formalin for histopathological examination. The tissues were embedded in paraffin and sectioned every 5 mm and the slides were examined by light microscope after haematoxylin and eosion staining.

### Statistical analysis

Significant (*P* < 0.05) differences between control and treated groups were compared by an unpaired *t*-test.

## Results

### Effects of SP on plasma cholesterol and triglycerides concentration in normal and HCD groups

**Fig 1:**
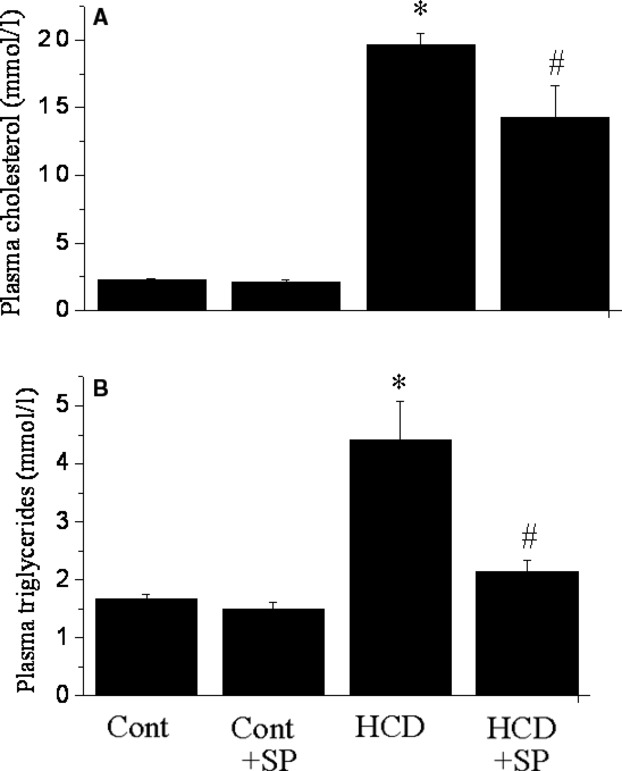
Effects of sarpogrelate on high cholesterol diet induced changes in plasma cholesterol and triglyceride levels in rabbits. Con, control; SP, sarpogrelate (5 mg/kg/day); HCD, high cholesterol (1%) diet for 90 days, *P < 0.05 *versus* cont; #*P* < 0.05 *versus* HCD. Each value is a mean ± SE of 4–12 animals in each group as indicated in the Methods section.

The data in Figure 1 show that rabbits fed HCD for 90 days demonstrated significant increase (8.6-fold) in the concentration of plasma cholesterol and 2.6-fold increase in the concentration of triglycerides compared with control group. Treatment with SP had no significant effect on plasma cholesterol and triglycerides in normal diet group. However, in HCD group, SP treatment significantly reduced the increases of plasma cholesterol and triglycerides concentrations compared with HCD group (Fig. 1).

### Effects of SP on plasma MDA and SOD concentrations in normal and HCD groups

**Fig 2:**
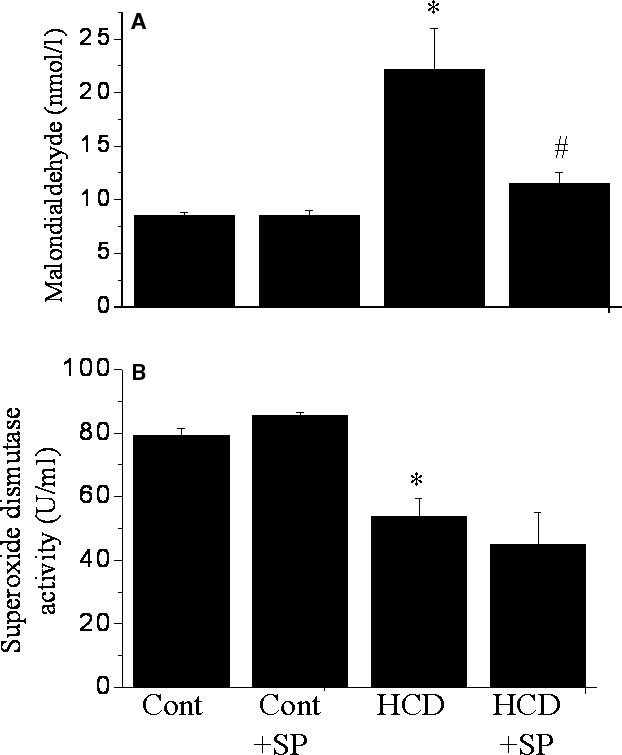
Effects of sarpogrelate on high cholesterol diet induced changes in plasma malondialdehyde and superoxide dismutase levels in rabbits. All abbreviations are same as in Fig. 1.

The effect of SP on oxidative stress was determined by measuring plasma MDA and SOD levels and the results are shown in Figure 2. The rabbits fed HCD for 90 days demonstrated a significant increase (2.6-fold) in the concentration of MDA and 32.9% decrease in the concentration of SOD compared with control group. Treatment with SP had no significant effect on plasma MDA and SOD levels in normal diet group. However, in HCD group, SP treatment reduced the increase of plasma MDA whereas treatment of animals with SP had no significant effect on the decrease of SOD level in the HCD group.

### Effects of SP on blood viscosity and plasma viscosity in normal and HCD groups

**Fig 3:**
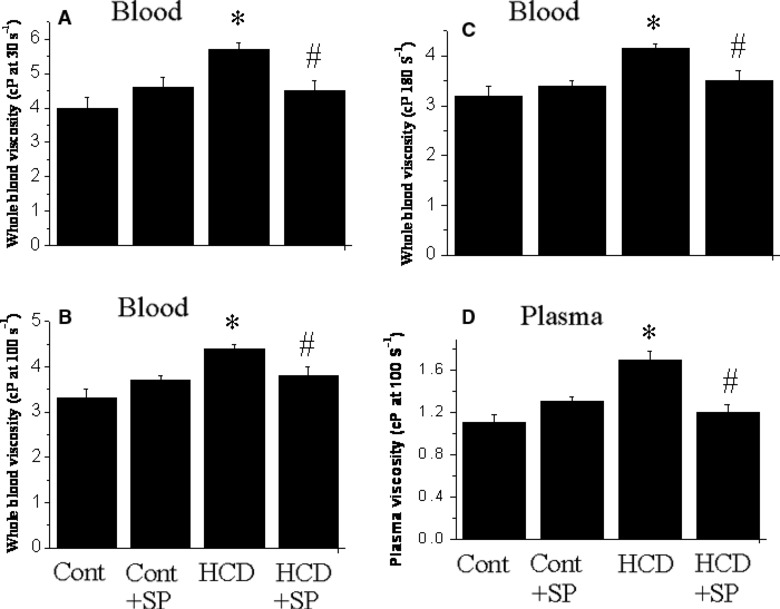
Effects of sarpogrelate on high cholesterol diet induced changes in whole blood and plasma viscosity in rabbits. cP – centipoise. All abbreviations are same as in Figure 1.

As whole blood and plasma viscosities reflect the intrinsic resistance to flow within blood vessels, the effects of SP on the blood and plasma viscosity were measured (Fig. 3). The rabbits fed HCD for 90 days showed increases of 24.6, 29.9, 32 and 32.16% in the blood viscosity at 9, 30, 100 and 180/S. shear rate compared with control group respectively. After SP treatment, blood viscosity were significantly lowered by 18.6, 13.6 and 14.2% at 30, 100, 180/S. shear rate respectively. The plasma viscosity was also significantly increased by 54.9% in HCD group compared with control group and this viscosity was decreased by 29% after SP treatment.

### Effects of SP on the formation of atherosclerotic plaque in aortic arch of rabbits fed HCD

**Fig 4:**
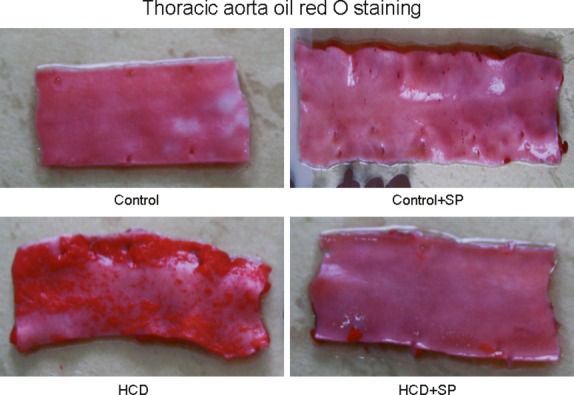
Representative illustration showing the effects of sarpogrelate on high cholesterol diet induced formation of atherosclerotic plaque in thoracic aorta of rabbits.

**Fig 5:**
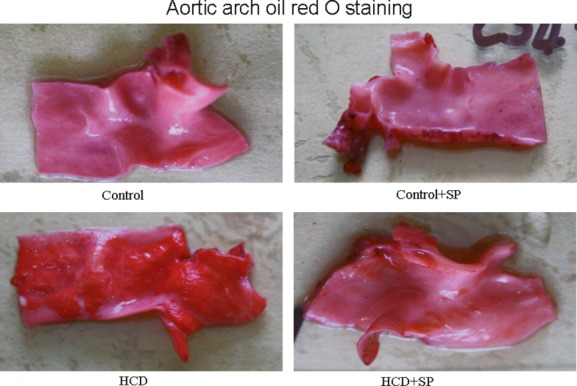
Representative illustration showing the effects of sarpogrelate on high cholesterol diet induced formation of atherosclerotic plaque in aortic arch of rabbits.

**Fig 6:**
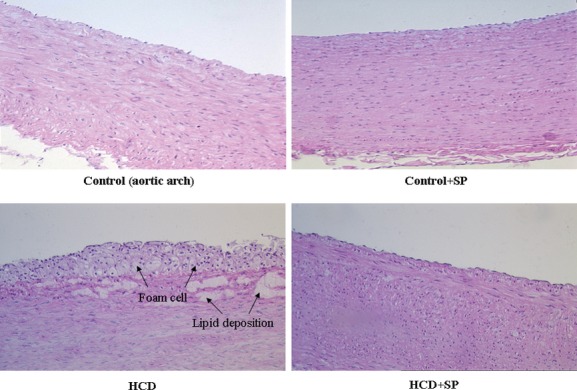
Representative illustration showing the effects of sarpogrelate on high cholesterol diet induced foam cells and lipid deposits in aortic arc.

As shown in Figures 4 and 5, the HCD group demonstrated heavy and rough surface staining in both thoracic aorta and aortic arch; the treatment with SP markedly reduced the staining areas in both parts of the aorta. In view of the fact that the atherosclerotic lesion in SP treated groups was not evident, semiquantative evaluation of morphological changes was not carried out. Histopathological examination of the aortic arch in normal rabbits revealed marked alterations in the vessel wall with the appearance of endothelial damage and multiple layers of thickened foam cells (Fig. 6). SP treatment provided protection against the high cholesterol-induced changes and showed a reduction in lesion area and foam cells.

## Discussion

Atherosclerosis is a major cause for many cardiovascular diseases like coronary heart disease and stroke. The first line of treatment for atherosclerosis is still lipid lowering drugs; however, the side effects of these agents with respect to liver function have limited their use [16]. On the other hand, anti-platelet and anti-oxidant therapies have recently gained much attention [17] and it is considered that drugs with properties for lowering blood cholesterol, reducing platelet aggregation and attenuating oxidative stress may become ideal and optimal choice for the treatment of atherosclerosis [17]. In fact, the results in the present study have demonstrated that SP has anti-oxidant and lipid lowering effects in HCD rabbits. Furthermore, SP was observed for the first time to reduce whole blood and plasma viscosity in HCD rabbits. It may be noted that 5-HT is released from activated platelets and has been reported to cause platelet aggregation and proliferation of VSMC [3, 18]. When platelets are activated by 5-HT or other stimuli, the platelet derived growth factor and lysophosphatidic acid (LPA) are released in the circulation. Lysophosphatidic acid stimulates the proliferation of VSMC and causes local inflammatory responses [19, 20]. Furthermore, 5-HT has a direct effect for increasing the intracellular concentration of free calcium in VSMC [9]; this positive feedback loop is believed to speed up the process of atherosclerotic plaque formation. Therefore, 5-HT receptor antagonists are expected to exert an anti-atherosclerosis effect. Hayashi *et al*. [10] have reported that SP produced a reduction in atherosclerotic plaque size which effect was potentiated by combination with vitamin E. In this study, we have observed that SP treatment attenuated plaque formation and significantly reduced serum concentrations of cholesterol and TG in HCD group. This observation is at variance with an earlier report indicating that SP has no effect on blood cholesterol and TG in diabetic patients [21]. These inconsistent results in these studies may be due to differences in duration and dose of drug treatment as well as species of experimental animals.

Hyperlipidaemia has been shown to increase blood viscosity and blood coagulation previously [22]. High blood viscosity, which is considered to increase the resistance between flowing blood and endothelial cells, may induce damage to the endothelial cells in the vasculature. Thus, more cholesterol is permitted to enter the wall of blood vessels and cause atherosclerotic plaque. It is pointed out that blood viscosity at lower shear rate (<30) is mainly influenced by the aggregation of red blood cells whereas at the higher shear rate (>100), the blood viscosity is mainly reflected by the deformability of red blood cells [23]. Our results show that SP reduced the whole blood viscosity in both lower and higher shear rate, indicating that SP may correct the disorder of red blood cells in the hyperlidaemic situation. The plasma viscosity, which is mainly affected by changes in cholesterol and TG concentrations, was also reduced by SP treatment in our study. As SP treatment did not affect the blood viscosity in normal rabbits, the effect of SP on blood viscosity appears to be secondary to its lipid lowering effect in animals fed HCD. These observations seem to suggest a novel mechanism involving changes in blood viscosity by SP for the treatment of atherosclerosis.

The oxidation of low density lipoproteins (Ox-LDL) is a critical step for the development of atherosclerosis [16]. Ox-LDL binds to its receptors on VSMC and endothelial cells and causes the proliferation of VSMC and local inflammation. Therefore, antioxidant therapy may be an efficient therapeutic approach to attenuate atherosclerosis in the hypercholesterolaemic patients [24]. It is noteworthy that Haidari *et al*. [25] have reported the augmentation of oxidative stress in the atherosclerosis-susceptible regions of the normal mouse aorta and free radical scavengers were shown to improve the endothelial dysfunction in coronary artery disease patients [26]. Furthermore, MDA, a membrane lipid peroxidation product, is considered to be a biomarker of oxidative stress in hyperlipidaemic patients [27, 28] and Yang *et al*. [29] have observed that oxidative stress associated with elevation of MDA is an early event in the pathogenesis of hyperlipidaemia. Although SP showed no effect on MDA level in normal rabbits, this treatment significantly reduced the elevated level of MDA in HCD group. These data suggest SP may be reducing oxidative stress in hypercholesterolaemic rabbits; however, it remains unclear whether this effect is secondary to the reduction of blood cholesterol or attenuation of the process of oxidation. As SOD activity was inhibited in HCD group and SP treatment was unable to reverse this effect, it is likely that the reduction of MDA by SP treatment is not through SOD pathways. Although it has been reported that SP increased SOD activity in diabetic rat and mouse models [30, 31], the inability of SP to reverse the SOD activity in HCD rabbits in our study may be due to differences in the animal species, as well as experimental models. In this regard, it is pointed out that SOD activity has been reported to be reduced in hyperlipidaemic patients showing high levels of TG and cholesterol [19, 32].

The present study has shown that the atherosclerotic plaque size and the number of foam cells were significantly reduced by SP treatment. From these observations, it is suggested that SP may be a preventative drug for atherosclerosis. Furthermore, on the basis of results described in this study, it appears that SP may exert anti-atherosclerotic action by reducing blood viscosity, oxidative stress and blood lipids. Such mechanisms of the SP action are complimentary to earlier observations that this agent was found to prevent proliferation of endothelial cells [30]. Origuchi *et al*. [33] have also reported that SP reduced the balloon-induced injury in hypercholesterolaemic rabbits. In addition, Kodama *et al*. [34] have demonstrated that SP attenuated intimal hyperplasia in rabbit vein graft. Whether this agent can prevent the progression of atherosclerosis or reverse this disease needs to be further investigated. Taken together, these findings support the view that SP may exert beneficial effects on the prevention of atherosclerosis by protecting the damage to endothelial cells, reducing thrombosis formation and platelet aggregation, as well as attenuating the migration of cholesterol and macrophage into the blood vessels.
